# Bacteriostatic activity and partial characterization of the bacteriocin produced by *L. plantarum* sp. isolated from traditional sourdough

**DOI:** 10.1002/fsn3.1890

**Published:** 2020-09-13

**Authors:** Mina Zangeneh, Sadegh Khorrami, Moj Khaleghi

**Affiliations:** ^1^ Department of Biology Faculty of Sciences Shahid Bahonar University of Kerman Kerman Iran

**Keywords:** antibacterial, bacteriocin, bacteriostatic, *Lactobacillusplantarum*

## Abstract

This study was aimed to isolate and partially characterizes the bacteriocin produced by an *L. plantarum* sp. isolated from traditional sourdough. The bacteriocin was partially purified, and after treating it with different harsh conditions, its antibacterial activity was evaluated against *L. monocytogenes* as an indicator. Also, the growth phase during which the bacteriocin is produced, and its mode of action, was examined. Finally, the molecular weight of this compound was evaluated by using SDS‐PAGE analysis. According to the results, this bacteriocin had a molecular weight well lower than 10 kDa that was mainly produced at the early stationary phase and reached its highest activity (3,200 AU/ml) at the same stage. It was tolerant toward a wide range of pH (2–10), temperatures (−20 to 120°C), and high concentrations of NaCl. Notably, the bacteriocin‐producing strain had proteolytic activity, while the bacteriocin produced by that showed resistance to proteolytic enzymes (pepsin, trypsin, and proteinase K). Also, it was revealed that the bacteriocin activity is mostly bacteriostatic so that it considerably inhibits pathogens’ growth, particularly *S. aureus*, *E. coli,* and *L. monocytogenes*. These characteristics prove that strain and its bacteriocin can be considered as one of the most promising agents to use in the food industry.

## Graphical abstract

The strain produces a low molecular weight bacteriocin (lower than 10 kDa) at the early log phase. This bacteriocin is highly stable against harsh conditions, such as low pH, and long term storage at low temperatures. What makes the compound unique is that it shows resistance to some proteinase. Furthermore, this compound has a bacteriostatic activity against pathogens.

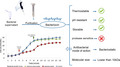

## 1 INTRODUCTION

Although rapid advances occurred in food processing technologies, preservation of nourishments for a long‐term period has always been one of the most challenging issues. Meanwhile, the demand for healthy, ready food is increasing in the modern world, putting the industry under pressure to meet these requirements and being profitable too. On the other hand, the bacterial resistance to antibiotics and dwindling ready foods' quality have raised many concerns. To solve these problems, many experts have recently focused on biological agents, such as plant extracts, animal‐derived enzymes, organic acids, and probiotic bacterial strains (Del Nobile, Lucera, Costa, & Conte, 2012). Among these strategies, probiotics are in a great position due to their positive influences on the function of the human gastrointestinal tract as well as their economic features. That is why during the last decades, a large number of academic investigations have been being conducted in this area.

The antimicrobial activity of probiotics is considered as an effective means to eliminate or inhibit pathogens (Dicks & Botes, 2010). Some probiotics through secreting antibacterial compounds, such as short‐chain fatty acids or hydrogen peroxide, and others by producing toxins, such as bacteriocins, combat with pathogens. Bacteriocins are well‐known antibacterial peptides/proteins synthesized by bacterial ribosomes, and either kill or inhibit the growth of closely related bacteria (Nes, Brede, & Diep, 2013). Bacteriocins produced by lactic acid bacteria (LAB) have been considered mainly due to their essential role in the fermentation process of food, the creation of a flavor, and food preservation (Costa et al.,; Gautam & Sharma, 2009). Furthermore, they are interesting due to their long history of use in food industries, and the generally regarded as safe (GRAS) and Qualified Presumption of Safety (QPS) status (Alvarez‐Sieiro, Montalbán‐López, Mu, & Kuipers, 2016; De Giani et al., 2019). The LAB family includes *Lactobacillus*, *Lactococcus*, *Leuconostoc*, *Pediococcus*, *Streptococcus*, *Aerococcus*, *Alloiococcus*, *Carnobacterium*, *Dolosigranulum*, *Enterococcus*, *Oenococcus*, *Tetragenococcus*, *Vagococcus,* and *Weissella* (Mokoena, 2017). Among these, *Lactobacillus* is the largest genus, including more than 100 species that are abundant in carbohydrate‐rich substances. Also, it is stated that *L. plantarum* is considered as one of the greatest and versatile species (Silva, Vitolo, González, de Oliveira, & S, 2014).

In contrast to all benefits to human health the probiotics possess, these days, there is an increasing concern about the resistance of the strains to antibiotics. Also, the quality of probiotics may well differ according to their source. Therefore, looking for high potential prebiotic strains is one of the essential branches of microbiology and food sciences. In our previous study, 16 *lactobacillus* strains were isolated from traditional sourdough, characterized, and their probiotic potential was described (Zangeneh, Khaleghi, & Khorrami, 2019). In the present study, we have focused on one of those strains that had the best antibacterial activity. Assuming this activity refers to the bacteriocin production by the strain, the supernatant of the bacterial culture was investigated and different features of the bacteriocin were examined.

## 2 MATERIAL AND METHODS

### 2.1 Bacterial strains and culture media

As our previous study revealed, SH1a, which showed a 95% similarity with *L. plantarum* sp. had the highest activity against pathogens. Over the present study, the intended strain was anaerobically grown under a 5% CO_2_ atmosphere in the MRS broth medium (Merck, Germany) at 37°C. To provide anaerobic conditions, the gas pack (Oxide Gas Generating Kit) was used. Also, the pathogens including *Staphylococcus aureus* PTCC 25,923, *Escherichia coli* PTCC 1,330, *Bacillus subtilis* PTCC 1,023, *Micrococcus luteus* PTCC 1,110, *Listeria monocytogenes* ATCC 35,152, and *Enterococcus faecalis* were purchased from Pasture Institute of Iran (Tehran, Iran) and were incubated in Trypticase Soy broth/agar (TSB/A, Merck, Germany) at 37°C. *L. monocytogenes* was considered as an indicator to evaluate the antibacterial activity of the bacteriocin (Zyl, Deane, & Dicks, 2019). Antibacterial tests were all done in the Muller Hinton medium (MH, Scharlau, Spain). The microplates used in this study were purchased from NEST, Wuxi, China.

### 2.2 Evaluation of the antibacterial activity of the isolated strain

The microtiter plate technique was used to test the antimicrobial activity of the bacteriocin‐producing Lactobacilli (Khaleghi, Khorrami, & Ravan, 2019). Briefly, 150 µl of the isolated strain was inoculated into 100 ml MRS broth and incubated for 24 hr. The overnight culture was centrifuged (9,000 g, 10 min, 4°C), and its supernatant was taken and divided into two portions. To neutralize/minimize the influence of organic acids, the pH of one of these proportions was adjusted to 6.5 using NaOH (1M), and the other remained unchanged (pH 4.4). After that, each sample was filtered through a 0.2 µm filter, and 100 µl of them was added to 150 µl MH broth containing 20 µl of overnight pathogens (0.5 McFarland). Finally, microplates were incubated, and after 24 hr, the activity of the supernatant against pathogens was assessed by evaluating the OD_600_ of each well. The well containing uncultured medium, as well as pathogens untreated with the supernatants, was considered as controls. Each experiment was done three times.

### 2.3 Extracellular proteolytic activity

Extracellular proteolytic activity of the bacteriocin‐producing strain was determined through the method described by Del Bello et al. with slight modifications (Dal Bello et al., 2012). Firstly, 5 µl of the strain suspension (0.5 McFarland) was dropped onto the surface of MRS agar containing 10% (w/v) skim milk powder (Sigma‐Aldrich) and incubated at 37°C for 24–48 hr. A clear zone around bacterial colonies indicates the proteolytic activity of the strain.

### 2.4 The bacterial growth curve and bacteriocin production

To determine the growth pattern of the strain, an overnight culture of the strain was adjusted to 0.5 McFarland standard, and 200 µl of it was inoculated to 200 ml MRS broth and incubated for 34 hr. During this period, every two hours, 5 ml of the culture was taken and their optical density (OD 600 nm) and pH were recorded. Meanwhile, to determine the growth phase during which the bacteriocin is produced, twofold serial dilutions of the supernatants derived from different incubation times were prepared and introduced to the *L. monocytogenes* fresh cultures, followed by 24 hr incubation. Next, OD 600 of the samples was measured and the bacteriocin activity was calculated as arbitrary units (AU) per ml using the equation below. One AU is defined as the reciprocal of the highest dilution resulting in a distinct growth inhibition on the indicator strain (Banerjee, Dora, & Chowdhury, 2013). Besides, the antibacterial activity of the bacteriocin taken from the 10‐hr and 20‐hr cultures (early log phase and early stationary phase, respectively) was compared by the well diffusion method (Khorrami, Kamali, & Zarrabi, 2020).

$$AU\;{\text{ml}}^{‐1}=reciprocal\;of\;highest\;twofold\;dilution\times 100$$

### 2.5 Partial purification of the bacteriocin

The bacteriocin purification was carried out according to the method described by Lohrasbi‐Nejad et al. with some modifications (Lohrasbi‐Nejad, Torkzadeh‐Mahani, & Hosseinkhani, 2016). At first, an overnight culture of the isolated strain was prepared and centrifuged at 9,000 g for 10 min. Next, 90% saturated ammonium sulfate was applied to the deposition of the existing total protein in 50 ml of the supernatant. The protein pellets were then dissolved in 2 ml sterile distilled water and desalted using a dialysis membrane with a molecular weight cut‐off of 3.5 KDa for 24 hr. After that time, the solution was passed through Sephadex column G2580‐50G (Sigma‐Aldrich, Germany). Finally, the obtained sample was lyophilized, resuspended in sterile distilled water (0.25 µg/ml), and stored at −20°C to further experiments.

### 2.6 Effects of pH, temperature, and storage time on the bacteriocin activity

To evaluate the bacteriocin sensitivity to various pH values, as previously described (Todorov, Favaro, Gibbs, & Vaz‐Velho, 2012), the bacteriocin solutions were adjusted to pH 2, 4, 6, 8, and 10 by adding HCl and NaOH (1M) and incubated for 2 hr at 37°C. After that, the antimicrobial activity of these samples was assessed by evaluating their effects on *L. monocytogenes,* as an indicator.

To determine the bacteriocin thermostability, solutions containing the bacteriocin were firstly treated at 60°C, 80°C, and 100°C for 15 and 30 min, and 121°C for 15 min. Then, the samples’ antimicrobial activity was measured through the microtiter plate assay (Hu et al., 2014).

The effect of storage at low temperature on the compound was studied by maintaining its solutions at 4°C for one week, and −20°C for six months. After these times, the antibacterial activity of the bacteriocin was tested against the indicator strain.

### 2.7 Effects of proteolytic enzymes on the bacteriocin

The impact of different proteolytic enzymes on the bacteriocin activity was investigated according to the method described previously (Bromberg, Moreno, Zaganini, Delboni, & Oliveira, 2004). Briefly, the following enzyme‐buffer solutions were prepared; trypsin (Sigma, Germany) in Tris‐HCl (40 mM), pH 8.2, pepsin (Merck, Germany) in 0.002 HCl, pH 2, and proteinase K (Sigma, Germany) in Tris‐HCl (10 mM). Then, 1 ml of these solutions was sterilized and mixed with 1 ml of the bacteriocin solution. The bacteriocin treated with the pure buffer was used as control. After 2 hr of incubation at 37°C, samples were heated in a boiling water bath for 5 min and then their antimicrobial activity was assessed.

### 2.8 The bacteriocin mode of action

To detect the mode of action of the bacteriocin against *L. monocytogenes*, at first, the growth curve of this strain was determined. After that, 100 ml of the TSB medium was inoculated with 100 µl of the strain (0.05 MacFarland) and incubated for 4 hr. Once the log phase of the strain started, 20 ml of the bacteriocin solution was added into the culture and the bacterial growth was monitored during a 1‐hr interval of time for 18 hr by using the colony count method. The *L. monocytogenes* cultures treaded by physiological serum (0.9% NaCl) were considered as controls. In a similar experiment, bacteriocin was added to the early stationary phase of the pathogen (8 hr after incubation) and subsequent changes were monitored through the colony count assay.

### 2.9 The molecular size of the bacteriocin

The molecular size of the partially purified bacteriocin was determined by using the Tricine‐SDS‐PAGE, according to the method described already (Gels, Schagger, & von Jagow, 1987; Reenen, 1998). Briefly, samples were dissolved in tricine loading buffer and loaded into the gel (Sigma‐Aldrich, Germany). After electrophoresis, the molecular weight of the bacteriocin was determined, following the gel staining with Coomassie blue R250 for 3 hr. A low molecular weight protein marker with sizes ranging from 10 to 72 kDa was used as a standard.

### 2.10 Statistical analysis

All experiments were carried out in triplicate. The results were expressed as mean and standard deviation (S.D). The values in the tolerance test were compared by one‐way ANOVA. *p* ˂ .05 was considered as a significant.

## 3 RESULTS

### 3.1 Elementary evaluation of Bacteriocinogenic LAB

**FIGURE 1:**
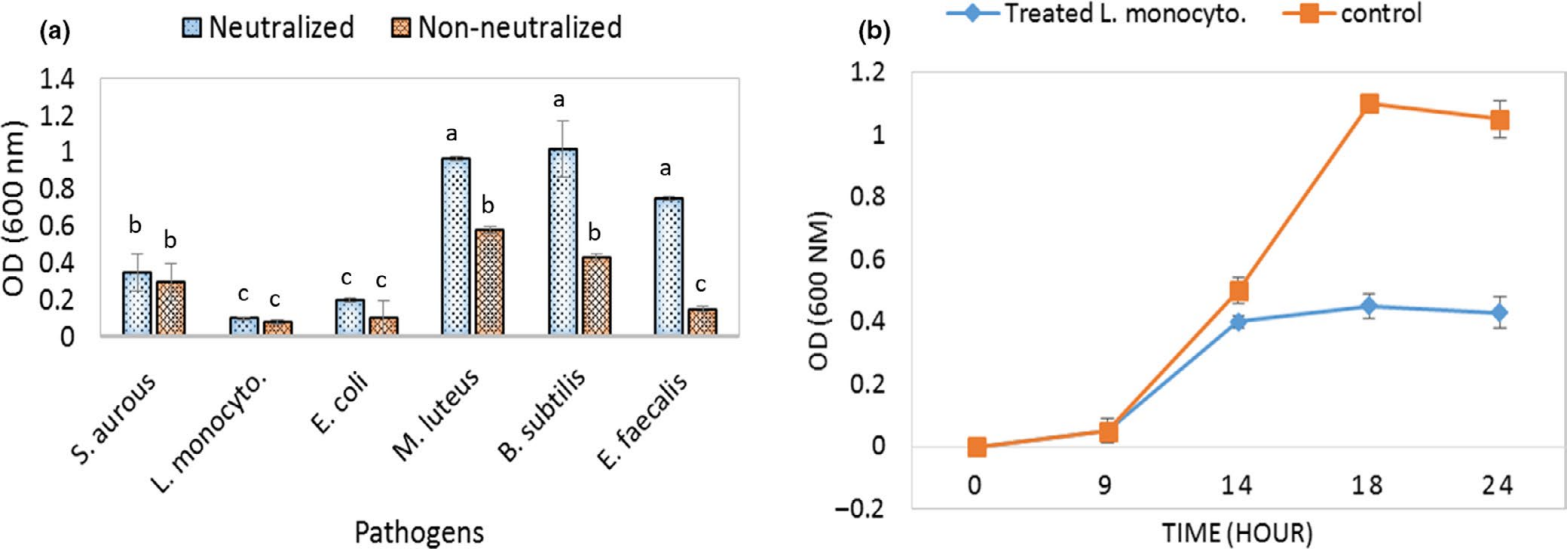
Antibacterial activity of the supernatant of *L. plantarum* sp. culture against pathogens (a). The OD_600_ of the *L. monocytogenes* treated and nontreated with the supernatant (b)

As it was expected, both non‐naturalized and naturalized supernatant of the isolated strain markedly inhibited the growth of the pathogens. The nan‐naturalized, however, showed slightly more activity. This effect on *B. subtilis* and *M. luteus* was less than others. While it was more significant on *L. monocytogenes*, *S. aureus,* and *E. coli*. Notably, the difference between the effect of neutralized and non‐neutralized supernatants on these strains was not significant (*P* ˂ .05) (Figure 1a), indicating the antibacterial activity is related to the bacteriocin production. Figure 1b shows how the neutralized supernatant affected *L. monocytogenes* during 24 hr incubation. It reduced the strain OD_600_ to 0.4, after 14 hr, and blocked the strain growth.

### 3.2 Extracellular proteolytic activity

**FIGURE 2:**
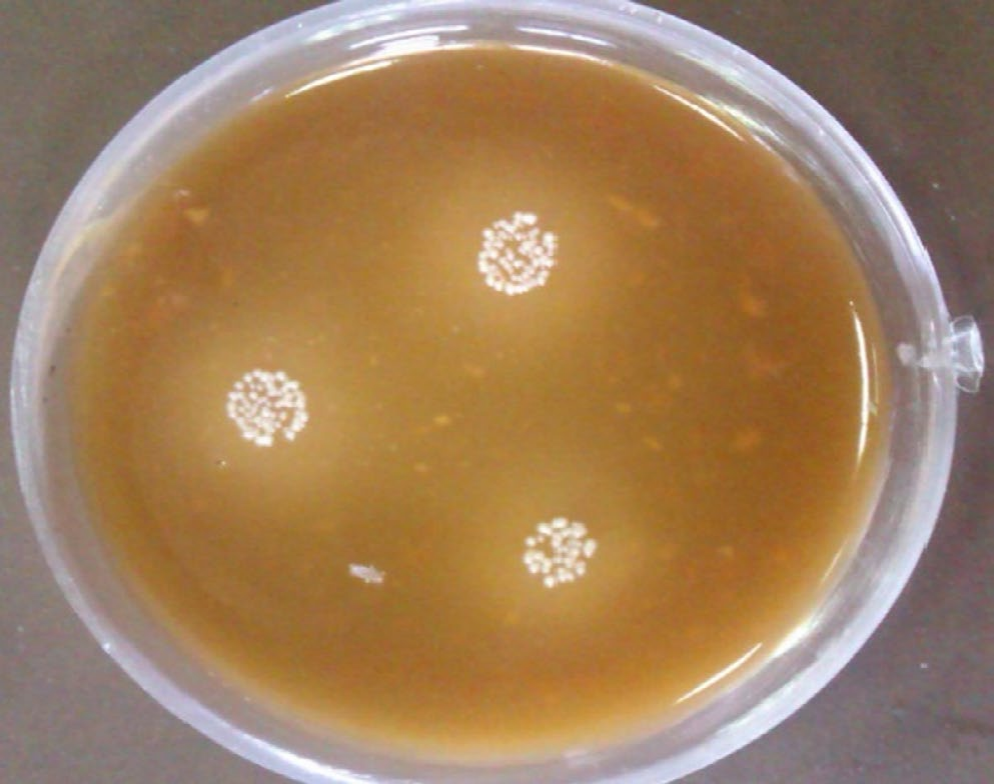
Proteolytic activity of the isolated strain. The zoon appeared around bacterial colonies indicates the proteolytic activity of the strain

The proteolytic activity of a strain can be determined by a clear zone that appears around the bacterial colony cultured on MRS/skimmed milk agar medium. As Figure 2 shows, after 24 hr, the diameter of the proteolytic zone expanded to 20 mm, confirming the isolated strain efficiently produces extracellular proteolytic enzymes (Lim, Foo, Loh, Mohamad, & Abdullah, 2019).

### 3.3 The bacterial growth curve and the bacteriocin activity

**FIGURE 3:**
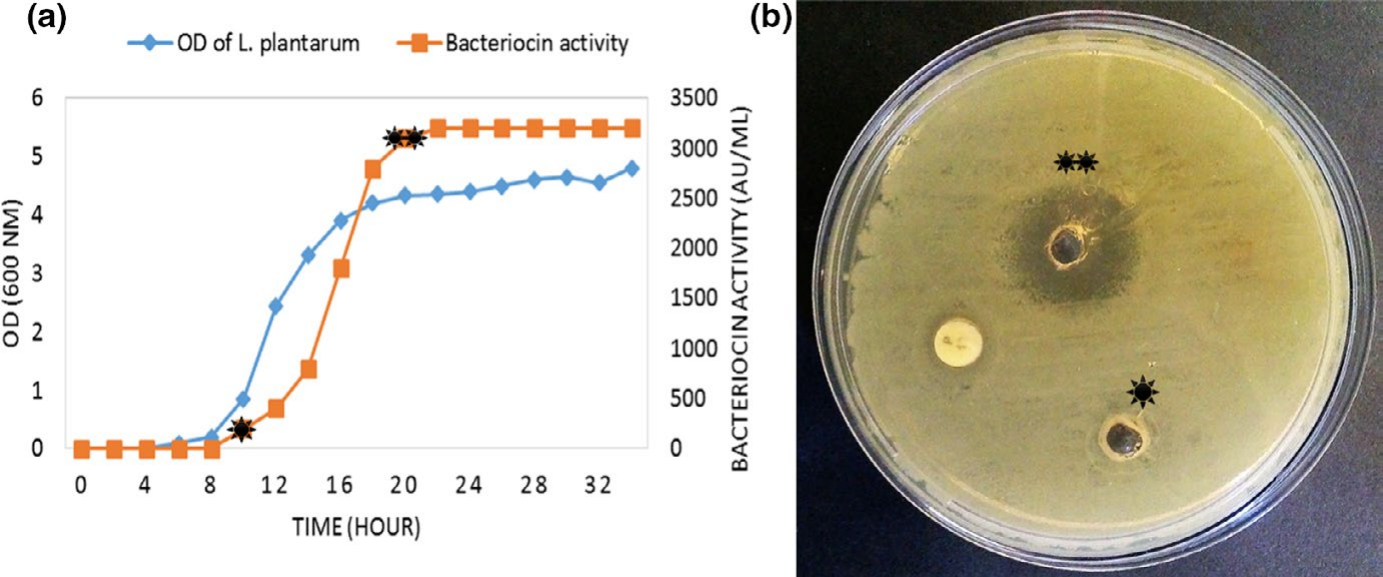
Growth curve of *L. plantarum* sp. as well as the bacteriocin activity (a). The antibacterial activity of the bacteriocin taken from the *L. plantarum* cultures, after 10 hr (*) and 20 hr (**) incubation, against *L. monocytogenes* (b)

The cell growth and bacteriocin production of the strain were monitored for 34 hr. According to the results, the strain swung to the exponential (log) phase 8 hr after its initial incubation, and it started its stationary phase after 20 hr since initial incubation. Bacteriocin production, also, started at the early log phase and reached its maximum level of 3,200 AU/ml at the early stationary phase (Figure 3a). Antibacterial activity of the bacteriocin taken from 10‐hr and 20‐hr cultures ware compared. As Figure 3b shows, the 10‐hr supernatant did not show any visible activity. For further studies, the supernatant was taken 20 hr after incubation.

### 3.4 Effects of pH, temperature, and storage time on the bacteriocin activity

Assessment of the bacteriocin activity after treatment with low pH and different temperatures revealed that this compound is entirely resistant to a wide range of pH and temperature (Table 1). So that it was stable after 2 hr incubation in pH 2 to 10. Likewise, the stability of the bacteriocin heated to 60, 80, and 100°C for 30 min and even 121°C for 15 min did not significantly change compared with the control/fresh bacteriocin. The bacteriocin activity, however, significantly declined after heating at 121°C for 30 min (Table 1). This activity remained stable during storage at 4°C for one week, and −20°C for six months.

**TABLE 1:** The effects of pH, storage conditions, temperature, and proteolytic enzymes on the antibacterial activity of the bacteriocin. These effects were evaluated based on the OD_600_ of *L. monocytogenes* as an indicator. (* significant data (*p* < .05))

pH	2				3			4			5			6			6.5			7			10	
OD_600_	0.54				0.54			0.53			0.54			0.54			0.56			0.55			0.54	
Storage conditions				Fresh bacteriocin								4°C, one week							−20°C, six months					
OD_600_				0.56								0.55							0.54					
Temperatures		25°C				60°C, 15 min	60°C, 30 min			80°C, 15 min			80°C, 30 min			100°C, 15 min		100°C, 30 min				121°C, 15 min		121°C, 30 min
OD_600_		0.50				0.52	0.53			0.53			0.54			0.55		0.55				0.56		0.58*
Enzymes			Control						Pepsin						Trypsin						Proteinase K			
OD_600_			0.54						0.54						0.52						0.53			

### 3.5 Effect of the proteolytic enzyme on the bacteriocin

Protease sensitivity assay revealed that this bacteriocin is considerably resistant to the enzymes (Table 1). According to the results, the difference between anti‐listeria activity of the bacteriocin treated with pepsin, trypsin, and proteinase K, and the control was insignificant (*p* ˂ .05).

### 3.6 The bacteriocin mode of action

**FIGURE 4:**
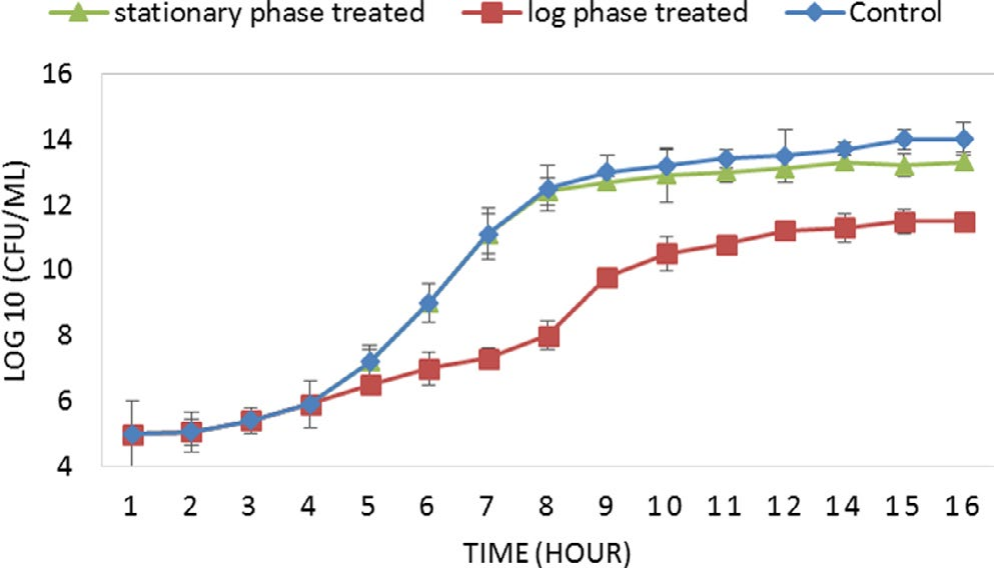
The bacteriocin's mode of action. The growth process of the pathogen considerably declines after introducing the bacteriocin into the early log phase of the strain that confirmed the bacteriostatic activity of the compound

As results have been shown in Figure 4, introducing the bacteriocin to the early log phase of *L. monocytogenes* significantly inhibited and delayed the growth process of the pathogen. It reduced the number of viable cells from 12.5 log CFU/ml to just 8 log CFU/ml after 8 hr (Figure 4). However, the bacteriocin introduced to the bacterial culture that had already started its stationary phase did not affect the bacterial growth considerably.

### 3.7 The bacteriocin size determination

**FIGURE 5:**
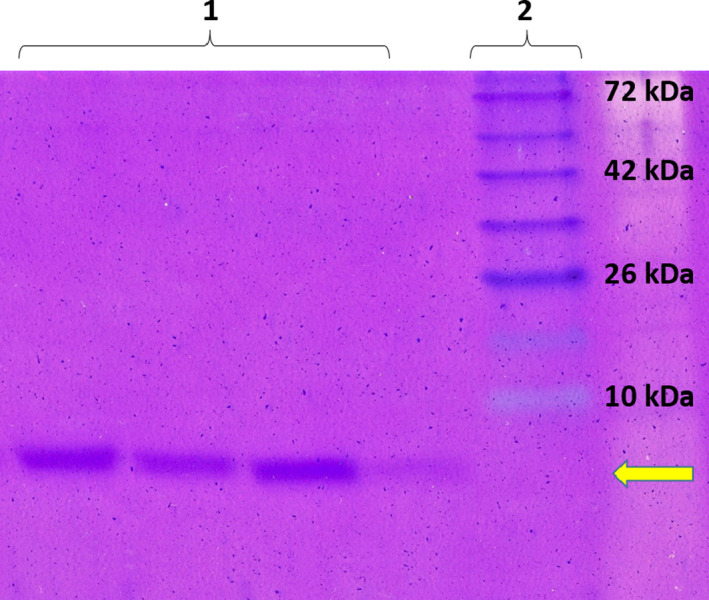
SDS‐PAGE analysis and size determination of the bacteriocin produced by the *L. plantarum* sp. strain. Lane 1: Three samples of the purified bacteriocin loaded on the gel; Lane 2: molecular weight marker

SDS‐PAGE analysis was done to investigate the bacteriocin molecular size. As Figure 5 shows, this bacteriocin in all three samples formed only one sharp band, which according to the ladder, the molecular weight of it was well below 10 kDa.

## 4 DISCUSSION

Bacteriocins produced by lactic acid bacteria are usually growth‐dependent metabolites, regulated by environmental conditions as well as the productive strain's growth phase (Papagianni & Papamichael, 2011; Sabo, Converti, Ichiwaki, & Oliveira, 2019). The findings of this study revealed that concerning the isolated strain (*L. plantarum*), the bacteriocin production initiates in the early log phase, and as the process goes on, this feature progresses up to 3,200 AU/ml, suggesting the bacteriocin is highly likely a primary metabolite. Also, Anastasiadou *et al*. have recently reported that Pediocin SA‐1 produced by *P. acidilactici* shows a similar manner. They have described this bacteriocin as a primary metabolite generated at the early phase of the bacterial growth (Anastasiadou, Papagianni, Filiousis, Ambrosiadis, & Koidis, 2008).

Furthermore, the bacteriocin characterized in the present study was very stable toward a wide range of temperatures, from −20°C to 120°C. Also, it maintained its function after long‐term storage (6 months). A similar compound formerly isolated from *L. brevis* by Banerjee *et al*. was stable for two months and 20 days when it was stored at −20°C and 4°C, respectively (Banerjee et al., 2013). Similarly, Plantaricin LPL‐1 produced by *L. plantarum* LPL‐1showed wide pH stability (2–10), as well as high thermal stability (121°C, 20 min) (Wang et al., 2018). The high stability of the bacteriocins makes them a robust agent that can be used in the food industry.

Up to now, many of the identified bacteriocins have been sensitive to proteolytic enzymes, and lose their function exposed to these enzymes. Quao et al., for example, have reported that enterocin TJUQ1 isolated from *E. faecium* TJUQ1 was inactivated after exposure to proteolytic enzymes. In contrast, it was not inactivated by lipase or amylase (Qiao, Du, Wang, Han, & Zhou, 2020). Likewise, it has been reported that a bacteriocin produced by *L. plantarum* isolated from traditional kombucha was sensitive to most proteases but not trypsin or pepsin (Pei et al., 2020). The compound introduced by Rasheed *et al*. missed all its activity in the presence of proteolytic enzymes (Proteinase K, pepsin, and trypsin) too. Not surprisingly, these compounds can quickly be degenerated by these enzymes because of their peptide nature (Rasheed et al., 2020).

However, the bacteriocin introduced in the present study was surprisingly resistant to trypsin, pepsin, and proteinase K. This feature has been reported in a few articles as well. Results of a study carried out by Singh *et al*., for instance, showed the antimicrobial performance of the bacteriocin characterized by them (laterosporulin) did not reduce after treating with proteolytic enzymes (pepsin, trypsin, chymotrypsin, proteinase K, and pronase E) (Singh, Sharma, Patil, & Korpole, 2012). Some scholars also believe the resistance of some low molecular weight peptides against some proteolytic enzymes is an expectable property (Ansari, Zohra, Tarar, Qader, & Aman, 2018; Singh, Sharma, Kumari, & Korpole, 2014; Yi, Luo, & Lü, 2018). It seems that the bacteriocin originated from *L. plantarum* sp. are intrinsically resistant to trypsin and pepsin. Furthermore, Class I and class II bacteriocins usually have spherical or amphiphilic alpha‐helix structures that it probably makes them immune toward proteolytic enzymes (Alvarez‐Sieiro et al., 2016).

Mode of action is one of the essential characters of a bacteriocin, allowing it to be applied efficiently. According to the results, when the bacteriocin was imposed on the *L. monocytogenes*, at the early exponential phase, not only the process was delayed, but also the number of surviving cells remarkably decreased. Moreover, imposing the bacteriocin at the early stationary phase resulted in a slight decrease in the bacterial CFU. These all indicate that the compound effectively inhibits the proliferation process of bacterial cells. Bacteriostatic activity of bacteriocins derived from *L. plantarum* sp. has already proofed by different researchers (Todorov, 2009; Ventura et al., 2015). In this regard, Pei *et al*. have recently reported these compounds increase the cell membrane permeability and cause the release of potassium ions, resulting in cell death (Pei et al., 2020). Also, it has been mentioned that interfering with the formation of the bacterial septum may also result in bacteriostatic action for some bacteriocins (Chikindas, Weeks, Drider, Chistyakov, & Dicks, 2018).

The physiochemical properties of the bacteriocin originated from *L. plantarum* isolated herein are very close to those of group IIa lactic acid bacteria (Wang et al., 2018). Even though the bacteriocin identified in this study is resistant to protease, almost all of them have similar features concerning their molecular weight, heat, and pH stability and sensitivity to proteolytic enzymes (Ghanbari, Jami, Kneifel, & Domig, 2013). The following characters are always typical of all members of class IIa bacteriocins; (a) their molecular weight is below 10 kDa. (b) They are undeniably active against *Listeria* spp. (c) they are resistant to high temperatures and pH value. (d) Their cystibiotic feature refers to the presence of at least one disulfide bridge, which is necessary for antibacterial activity (Belguesmia, Naghmouchi, Chihib, & Drider, 2011; Cotter, Hill, & Ross, 2005; Ghanbari et al., 2013).

## 5 CONCLUSION

Herein, a bacteriocin‐producing lactic acid strain was studied. It was revealed that the strain produces a low molecular weight bacteriocin (lower than 10 kDa) at the early log phase, and actively secrets it into the medium. This bacteriocin is highly stable against harsh conditions, such as low pH, and long‐term storage at low temperatures. What makes the compound unique is that it shows resistance to some proteinase. Furthermore, this bacteriocin has bacteriostatic activity against pathogens. These characteristics prove the strain and its bacteriocin as one of the most promising bioagents to be used in the food industry.

## CONFLICTS OF INTEREST

The authors declare that they have no conflicts of interest.

## **AUTHORS'** **CONTRIBUTION**

*Mina Zangeneh*: involved in conceptualization‐equal, investigation‐equal, methodology‐equal. *Sadegh Khorrami*: involved in conceptualization‐equal, writing‐original draft‐equal, writing‐review & editing‐equal. *Moj Khaleghi*: involved in resources‐equal and supervision‐equal, writing‐review & editing‐equal.

## ETHICAL APPROVAL

This article contains no studies with human participants or animals performed by any of the authors.

## Data Availability

The data that support the findings of this study are available on request from the corresponding author. The data are not publicly available due to privacy or ethical restrictions.
